# Supplementary material for: Withania somnifera Extracts Promote Resilience against Age-Related and Stress-Induced Behavioral Phenotypes in Drosophila melanogaster; a Possible Role of Other Compounds besides Withanolides
Source: Nutrients. 2022 Sep 22;14(19):3923. doi: 10.3390/nu14193923 (PMC9573261; doi:10.3390/nu14193923)
Supplement: Supplementary file 1 [file nutrients-14-03923-s001.zip › nutrients-1866469-supplementary.pdf]

Supplementary material

BEN-WSAq-9	0.5 mg/g				2 mg/g				5 mg/g			
	Day 0		Day 7		Day 0		Day 7		Day 0		Day 7	
	Mean	SEM	Mean	SEM	Mean	SEM	Mean	SEM	Mean	SEM	Mean	SEM
Withanolide B (ng/g)	ND		ND		3.09	0.19	4.33	0.17	11.47	0.46	14.69	0.41
Withanolide A (ng/g)	714.87	44.44	1008.31	31.82	2557.44	99.25	3216.62	100.49	7264.65	262.11	8898.27	314.25
12-Deoxywithastramonolide (ng/g)	31.58	2.85	41.20	1.51	108.44	3.76	133.20	3.50	299.71	11.35	357.40	16.80
Withaferin A (ng/g)	7.20	2.08	5.22	0.43	31.09	10.67	9.29	0.64	27.60	6.49	22.00	1.89
Withanone (ng/g)	514.69	26.25	721.00	16.59	1877.76	42.25	2358.51	39.21	5213.87	143.34	6588.02	147.77
Withanoside V (ng/g)	34.71	4.59	47.51	0.87	126.04	3.54	166.51	2.92	392.78	14.42	436.73	12.65
Withanoside IV (ng/g)	109.11	9.80	146.42	4.54	412.87	11.65	514.27	10.22	1196.11	47.18	1387.78	38.66

Table S1. Measurement of phytochemical markers in *Drosophila* food supplemented with WSAq-9. WS phytochemical marker levels (ng/g) in food at the day of preparation (day 0) and when kept for 7 days at 25°C were measured. Food samples from three different preparations were analyzed for each concentration and day.

BEN-WSE-2	0.5 mg/g			
	Day 0		Day 7	
	Mean	SEM	Mean	SEM
Withanolide B (ng/g)	4.60	0.44	7.93	0.23
Withanolide A (ng/g)	3447.93	210.26	4830.63	308.72
12-Deoxywithastramonolide (ng/g)	90.83	5.82	121.17	5.19
Withaferin A (ng/g)	54.33	18.01	24.70	3.56
Withanone (ng/g)	2826.63	128.33	4018.33	199.89
Withanoside V (ng/g)	220.77	13.37	309.40	4.34
Withanoside IV (ng/g)	651.20	39.31	938.90	9.61

Table S2. Measurement of phytochemical markers in *Drosophila* food supplemented with WSE-2. WS phytochemical marker levels (ng/g) in food at the day of preparation (day 0) and when kept for 7 days at 25°C were measured. Food samples from three different preparations were analyzed for each day.

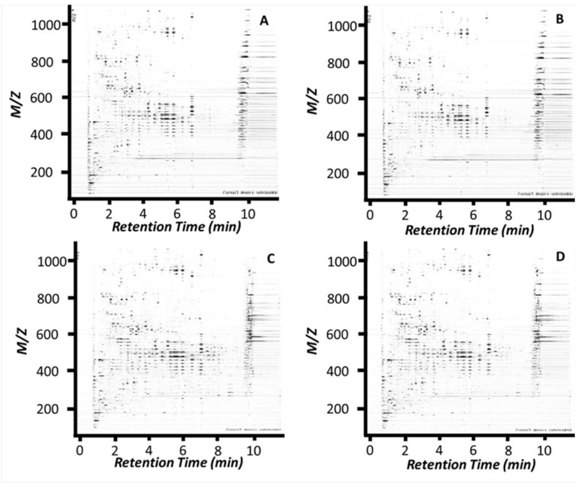

Figure S1. LC-HRMS (ESI+) fingerprints A) WSAq2, B) WSAq9, C) WSE2 and D) WSE5. LC-HRMS fingerprints were acquired for digital archiving of extracts to accompany voucher samples.

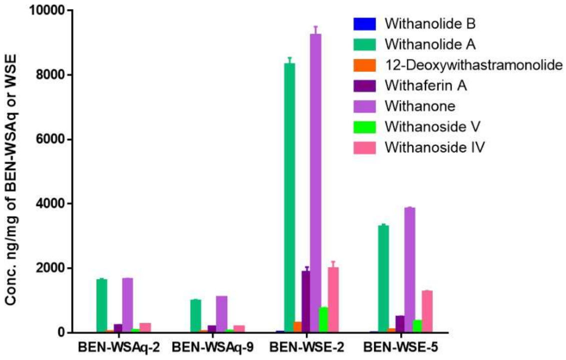

Figure S2. Levels of WS phytochemical markers (ng/mg of extract powder) measured in WSAq and WSE using LC-MRM-MS (as described under 2.3.1.). Analysis was performed in triplicate (N=3); mean +/- STD

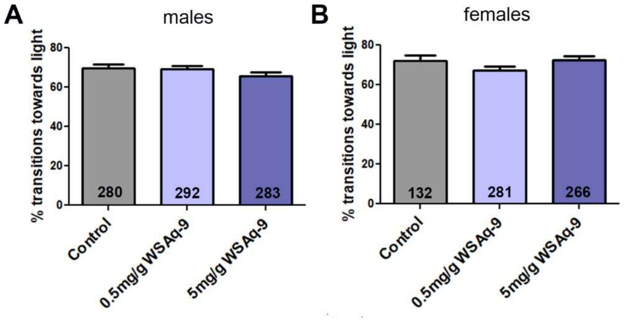

Figure S3. Treatment with WSAq-9 had no effect in young flies. Feeding flies after eclosion for 14d with 0.5mg/g or 5mg/g of WSE-2 before testing did not affect the phototaxis performance of males (A) or females (B). Kruskal-Wallis ANOVA with built-in Bonferroni post-hoc was used for statistical analyses. The number of tested flies is given in the bars.

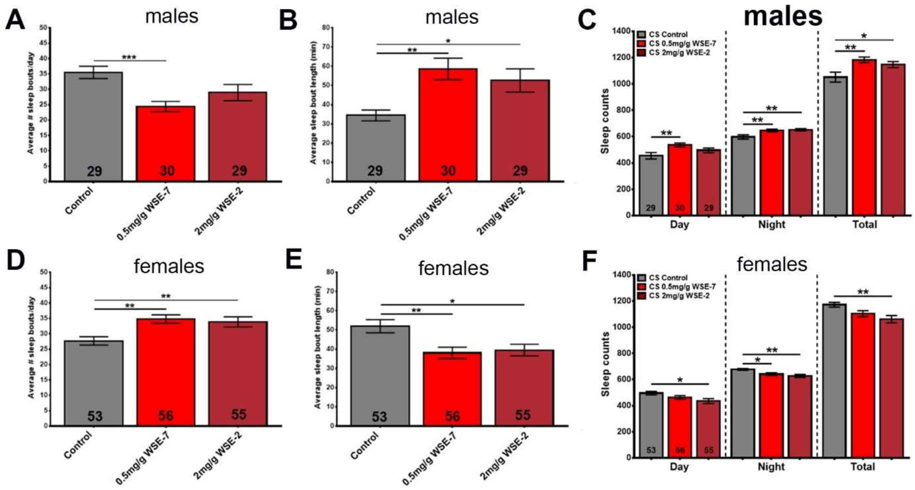

Figure S4. Continuous Continuous feeding of WSE-2 improves sleep in males but not in females. Males show a reduction in sleep bout number that is significant with 0.5mg/g WSE-2 (A). This is accompanied by a significant increase in sleep bout length at both concentrations (0.5mg/g and 2mg/g, B). WSE-2 treatment also increases nighttime sleep and total daily time spent asleep (C). Females do show an increase in sleep bout number (D) and a decrease in sleep bout length at both concentrations of WSE-2 (E). Both concentrations decreases nighttime sleep and total time spent asleep (F). A one-way ANOVA with Dunnett's multiple comparisons test was used and the number of analyzed flies is given in the bars. * $p < 0.05$, ** $p < 0.01$ compared to controls on regular food.
